# Measuring mental health and wellbeing outcomes for children and adolescents to inform practice and policy: a review of child self-report measures

**DOI:** 10.1186/1753-2000-8-14

**Published:** 2014-04-29

**Authors:** Jessica Deighton, Tim Croudace, Peter Fonagy, Jeb Brown, Praveetha Patalay, Miranda Wolpert

**Affiliations:** 1 Evidence Based Practice Unit (EBPU), UCL and the Anna Freud Centre, 21 Maresfield Gardens, London NW3 5SD, UK; 2 Mental Health and Addiction Research Group (MHARG), HYMS and Department of Health Sciences, Room 204, 2nd Floor (Area 4) ARRC Building, University of York, York, Heslington YO10 5DD, UK; 3 Research Department of Clinical, Educational and Health Psychology, UCL, Gower Street, London WC1E 6BT, UK; 4 Center for Clinical Informatics, 2061 Murray Holliday Blvd, Salt Lake City, UT 84117, USA

**Keywords:** Mental health outcomes, Measurement, Children, Child mental health services, Patient reported outcome measures

## Abstract

There is a growing appetite for mental health and wellbeing outcome measures that can inform clinical practice at individual and service levels, including use for local and national benchmarking. Despite a varied literature on child mental health and wellbeing outcome measures that focus on psychometric properties alone, no reviews exist that appraise the availability of psychometric evidence *and* suitability for use in routine practice in child and adolescent mental health services (CAMHS) including key implementation issues. This paper aimed to present the findings of the first review that evaluates existing broadband measures of mental health and wellbeing outcomes in terms of these criteria. The following steps were implemented in order to select measures suitable for use in routine practice: literature database searches, consultation with stakeholders, application of inclusion and exclusion criteria, secondary searches and filtering. Subsequently, detailed reviews of the retained measures’ psychometric properties and implementation features were carried out. 11 measures were identified as having potential for use in routine practice and meeting most of the key criteria: 1) Achenbach System of Empirically Based Assessment, 2) Beck Youth Inventories, 3) Behavior Assessment System for Children, 4) Behavioral and Emotional Rating Scale, 5) Child Health Questionnaire, 6) Child Symptom Inventories, 7) Health of the National Outcome Scale for Children and Adolescents, 8) Kidscreen, 9) Pediatric Symptom Checklist, 10) Strengths and Difficulties Questionnaire, 11) Youth Outcome Questionnaire. However, all existing measures identified had limitations as well as strengths. Furthermore, none had sufficient psychometric evidence available to demonstrate that they could reliably measure both severity and change over time in key groups. The review suggests a way of rigorously evaluating the growing number of broadband self-report mental health outcome measures against standards of feasibility and psychometric credibility in relation to use for practice and policy.

## Introduction

There is a growing number of children’s mental health and wellbeing measures that have the potential to be used in child and adolescent mental health services (CAMHS) to inform individual clinical practice e.g. [1], to provide information to feed into service development e.g. [2] and for local or national benchmarking e.g. [3]. Some such measures have a burgeoning corpus of psychometric evidence (e.g., Achenbach System of Empirically Based Assessment, ASEBA [4]; the Strengths and Difficulties Questionnaire, SDQ [5,6]) and a number of reviews have usefully summarized the validity and reliability of such measures [7,8]. However, it is also vital to determine which measures can be feasibly and appropriately deployed in a given setting or circumstance [8]. While some attempt has been made to identify measures that might be used in routine clinical practice [9] no reviews have evaluated in depth both the psychometric rigor and the utility of these measures.

National and international policy has focused on the importance of the voice of the child, of shared decision making for children accessing health services, and of self-defined recovery [10-13]. This policy context gives a clear rationale for the use of self-report measures for child mental health outcomes. Further rationale is provided by the costs of administration and burden for other reporters. For example, typical costs for a 30 minute instrument to be completed by a child mental health professional could be as much as £30 (clinical psychologist, £30.00; mental health nurse, £20.00; social worker, £27.00; generic CAMHS worker, £21.00; [14]). However, research has indicated that, due to their difficulties with reading and language and their tendencies to respond based on their state of mind at the moment (rather than on more general levels of adjustment), children may be less reliable in their assessments of their own mental health, and there is evidence of under-reporting behavioral difficulties [15,16]. Yet, there is increasing evidence that even children with significant mental health problems understand and have insight on their difficulties and can provide information that is unique and informative. Providing efforts are made to ensure measures are age appropriate (in terms of presentation and reading age), young children can be accurate reporters of their own mental health [17-19]. Even in the case of conduct problems, which are commonly identified as problematic for child self-report, evidence suggests that the use of age appropriate measures can yield valid and reliable self-report data [20]. In particular, a number of interactive, online self-report measures have been developed e.g., Dominic interactive; and see [17,21], which appear to elicit valid and reliable responses from children as young as eight years old.

Assessing mental health outcome measures for use in CAMHS also requires consideration of how outcomes should be compared across services. While more specific measures may provide a more detailed account of specific symptomatology, and may be more sensitive to change, they raise challenges in making comparisons across cases or across services where differences in case mix from one setting to the next are likely. Broad mental health indicators in contrast are designed to capture a constellation of the most commonly presented symptoms or difficulties and, therefore, are of relevance to most of the CAMHS population. They also reduce the need to isolate particular presenting problems at the outset of treatment in order to capture baseline problems to assess subsequent change against – a difficult task in the context of changing problems or situations across therapy sessions [22,23]. Associated with breadth of the measure is the issue of brevity; even if costs associated with clinician reported measures are avoided, long child self-report measures are likely to either erode clinical time where completed in clinical sessions or present barriers to completion for children and young people when administered outside sessions [22].

The current study is motivated by the argument that challenges to valid and reliable measurement of child mental health outcomes for those accessing services do not simply relate to the selection of a psychometrically sound tool; issues of burden, financial cost and suitability for comparison across services are huge barriers to successful implementation. Failure to grapple with such efficacy issues is likely to lead to distortions (based on attrition, representativeness and perverse incentives) in the yielded data. This review places particular importance on: 1) measures that cover broad symptom and age ranges, allowing comparisons between services, regions and years; 2) child self-report measures that offer more service user oriented and feasible perspective on mental health outcomes; 3) measures with a range of available evidence relating to psychometric properties, and 4) the resource implications of measures (in terms of both time and financial cost).

## Review

### Method

**Figure 1:**
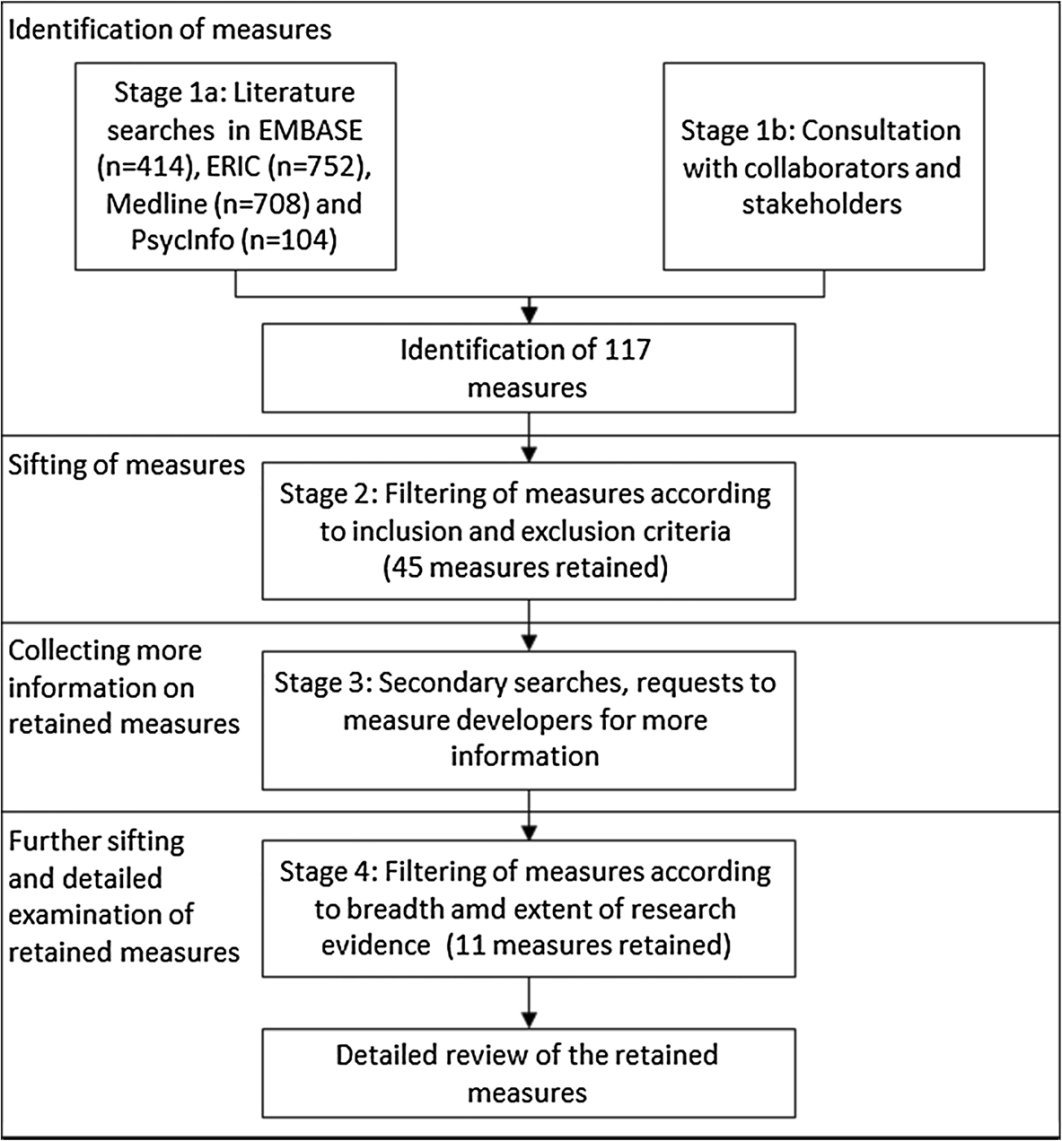
Flow diagram summarizing the review process.

The review process to identify and filter appropriate measures consisted of four stages, summarized in Figure 1.

The review was carried out by a team of four researchers, one review coordinator and an expert advisory group (five experts in child mental health and development, two psychometricians, three educational psychology experts and one economist). The search strategy, and inclusion and exclusion criteria were developed and agreed by the expert advisory group. Searches in respective databases and filtering were carried out by the researchers and review coordinator. Any ambiguous cases were taken to the expert advisory group for discussion.

#### Stage 1: Setting review parameters, literature searching and consultation

The key purpose of this review was to identify measures that could be used in routine CAMHS in order to inform service development and facilitate regional or national comparison. Because any outcome data collected for these purposes would need to be aggregated to the service level in sufficient numbers to provide reliable information, and would need to allow comparison across services and across years, only measures that cover broad symptom and age ranges were considered. The review focused on measures that included a child self-report version. This was partly because of the cost and burden implications associated with other reporters, especially clinicians, but also because of the recent emphasis on patient reported outcome measures e.g. [11] and evidence that, where measures are developed specifically to be child friendly, children can be accurate reporters of their own mental health e.g. [17,19]. The review focused on measures that had strong evidence of good psychometric properties and also took account of the resource implications associated with the measures (in terms of both time and financial cost).

#### Developing the search terms

For the purposes of this review, child mental health outcome measures were included if they sought to provide measurement of mental health in children and young people (up to age 18). To capture this, search terms were developed by splitting ‘child mental health outcomes measure’ into three categories: ‘measurement’, ‘mental health’ and ‘child’. A list of words and phrases reflecting each category was generated (see Table 1).

**Table 1:** Search terms

**Categories**	**Related words and phrases**
Measures and approaches to measurement	Measure; questionnaire; survey; checklist; check list; tool; rating scale; scale; repository
Mental health and psychological wellbeing	Mental health; quality of life; psychological adjustment; behaviour problems; emotional problems; mental illness; mental disorder; psychiatric disorder; behavioural and emotional difficulties; social difficulties; social and behavioural difficulties; conduct problems; internalising; externalising; depressive symptoms; antisocial; self-esteem; pride; prosocial behaviour; sense of belonging; hopefulness; wellbeing; positive self-regard; aggression; anxiety; depression; mood; feeling
Children	Children; adolescents; paediatrics

#### Search of key databases

Search terms were combined using ‘and’ statements to carry out initial searches focused on 4 key databases: EMBASE, ERIC, MEDLINE and PsychInfo. Searches resulting in over 200 papers were subjected to basic filtering using the following exclusion criteria: 1) the title made it clear that the paper was not related to children’s mental health outcome measures; or 2) the paper was not in English.

The remaining papers were further sorted based on more specific criteria. Papers were removed if:

• No child mental health outcome measure was mentioned in the abstract;

• The measure indicated was too narrow to provide a broad assessment of mental health;

• They referred to a measure not used with children;

• They were not in English;

• They were a duplicate;

• The measure was used solely as a tool for assessment or diagnosis.

A list of identified measures was collated from the papers that were retained.

#### Consultation with collaborators and stakeholders

In order to identify other relevant measures, consultation with two key groups about their knowledge of other existing mental health measures was conducted: 1) the experts in child and adolescent psychology, education and psychometrics from the research group, 2) child mental health practitioners accessed via established UK networks (from which 58 practitioners responded). At the completion of Stage 1, 117 measures had been identified.

#### Stage 2: Filtering of measures according to inclusion and exclusion criteria

In order to determine which of these measures were to be considered for more in-depth review, inclusion and exclusion criteria were established.

#### Inclusion criteria

A questionnaire or measure was included if it:

• Provided measurement of broad mental health and/or wellbeing in children and young people (up to age 18), including measures of wellbeing and quality of life;

• Was completed by children;

• Had been validated in a child or adolescent context.

#### Exclusion criteria

A questionnaire or measure was excluded if it:

• Was not available in English;

• Concerned only a narrow set of specific mental disorders or difficulties;

• Could only be completed by a professional;

• Took over 30 minutes to complete;

• Primarily employed open-ended responses;

• Used an age range that was too narrow (e.g. only for preschoolers);

• Had not been used with a variety of populations.

Applying these criteria generated a list of 45 measures see [24].

#### Stage 3: Secondary searches

The initial searches provided preliminary information on these 45 measures. However, secondary searches on these measures were conducted in order to gather further information about:

• Psychometric properties;

• Symptoms or subscales covered;

• Response format;

• Length;

• Respondent;

• Age range covered;

• Number of associated published papers;

• Settings in which the measure has been used.

Information on specific measures was sought from the following sources (in order of priority): measure manuals, review papers, published papers (prioritizing the most recent), contact with the measure developer(s), other web-based sources. Measures were excluded if no further information about them could be gathered from these sources.

#### Stage 4: Filtering of measures according to breadth and extent of research evidence

After collecting this information, the measures were filtered based on the quality of the evidence available for the psychometric properties. Measures were also removed at this stage if it transpired they were earlier versions of measures for which more recent versions had been identified. The original inclusion and exclusion criteria were also maintained. In addition, the following criteria were now applied:

1. Heterogeneity of samples – the measure was excluded if the only evidence for it was in one particular population, specifically children with one type of problem or diagnosis (e.g., only those with conduct problems or only those with eating disorders).

2. Extent of evidence – the measure was retained only if it had more than five published empirical studies that reported use with a sample or if psychometric evidence was available from independent researchers other than the original developers.

3. Response scales – the measure was retained only if its response scale was polytomous; simple yes/no checklists or visual analogue scales (VAS) were excluded.

These relatively strict criteria were used to identify a small number of robust measures that are appropriate for gauging levels of wellbeing across populations and for evaluating service level outcomes. After these criteria were applied, the retained measures were subjected to a detailed review of implementation features (including versions, age range, response scales, length and financial costs) and psychometric properties. The range of psychometric properties considered included content validity, discriminant validity, concurrent validity, internal consistency and test-retest reliability. We also considered whether the measure had: undergone analysis using item response theory (IRT) approaches (including whether the measure had been tested for bias or differential performance in different UK populations); evidence of sensitivity to change; or, evidence of being successfully used to drive up performance within services.

## Results

The application of the criteria outlined resulted in the retention of 11 measures. The implementation features and psychometric properties of these measures are outlined in Tables 2 and 3.

**Table 2:** Implementation features of the 11 measures identified after stage 4

**Measure**	**Scales and subscales**	**Versions**	**Age**	**Length/time to complete**	**Response scales**	**Cost associated with use?**	**Other languages**
1. Achenbach System of Empirically Based Assessment (ASEBA)	Covers the following domains: anxious/depressed, withdrawn/depressed, somatic complaints, social problems, thought problems, attention problems, rule-breaking behaviour and aggressive behaviour. Also summed into internalising and externalising subscales	Child Behaviour Check List (CBCL, parent/carer report); Teacher Report (TRF); Youth Self-Report (YSR)	TRF and CBCL: 1.5-5yrs and 6-18 years	YSR = 105 items, 15mins	0, 1, 2 (always, sometimes, never)	Yes	A range of versions have been translated into over 80 different languages
				TRF = 120 items, 15 minutes			
			YSR: 11-18 years				
				CBCL = 120 items, 15 minutes			
2. Beck Youth Inventories (BYI)	5 child self-report inventories: depression inventory, anxiety inventory, anger inventory, disruptive behavior inventory, self-concept inventory	All self-report	7-18 years	5 inventories, each with 20 questions, 5 minutes per inventory.	0, 1, 2, 3 (never, sometimes, often, always).	Yes	English
3. Behavior Assessment System for Children (BASC)	Covers the following: hyperactivity, aggression, conduct problems, anxiety, depression, somatization, attention problems, learning problems, withdrawal, atypicality, adaptability, leadership, social skills and study skills	Teacher Report Scale (TRS) - 14 scales; Parent Report Scale (PRS) - 13 scales; Self-report of Personality (SRP) - 14 scales	PRS and TRS, 3 age groupings: preschool (ages 2 years to 5 years), child (ages 6 years to 11 years), and adolescent (ages 12 years to 21 years	PRS = 134-160 items (10-20 minutes to complete)	PRS, TRS & SRP: 4 point scale (never, sometimes, often and almost always) SRP also has some true/false	Yes	English and Spanish
				TRS = 100-139 items (10-15 minutes)			
				SRP = 139-185 (20-30 minutes)			
4. Behavioral and Emotional Rating Scale (BERS)	6 factors: interpersonal strength, family involvement, intrapersonal strength, school functioning, affective strength, career strength (CS is new to BERS-2)	Teacher rating scale (TRS); Parent rating scale (PRS); Youth Rating Scale (YRS)	5-18 years	52 items in parent/carer and teacher scales - 10 minutes	0, 1, 2, 3 (not at all like the child; not like the child; like the child; very much like the child).	Yes	English and Spanish
				Eight open-ended questions			
5. Child Health Questionnaire (CHQ)	Parent 50 - 14 concepts (12 scales and 2 single items)	Parent/carer and child report versions	Self- report: 10+ years	Self-report: 87 items	5 point scale, labels vary	Free for research purposes	Some versions have been translated into over 70 different languages
	Parent 28 - 14 concepts (12 scales and 2 single items)		Parent/carer report: 5-18 years	Parent/carer report: 28 or 50			
	Child form- 12 concepts (10 scales and 2 items)						
	Including physical functioning, bodily pain, general health perceptions, self-esteem, mental health, behaviour						
6. Child Symptom Inventories (CSI)	Covers a range of disorders such as ADHD, Oppositional Defiant Disorder, Conduct Disorder, Generalized Anxiety Disorder, Obsessive-Compulsive Disorder, Specific Phobia, Major Depressive Disorder and more.	Parent/carer, teacher and child self-reports.	ECI-4: 3-5years	Between 77 and 108 items depending on version and reporter	4-point response scale, indicating how often the symptom is observed	Yes	Parent/carer checklist available in 14 languages
		Parent/carer and teacher: ECI-4 (Early Childhood Inventory); CSI-4 (Child Symptom Inventory);	CSI-4: 5-12 years				
			ASI-4: 12-18 years				
			YI-4: 12-18 years				
		ASI-4 (Adolescent Symptom Inventory).					
		Self-report: YI-4 (Youth’s Inventory)					
7. Health of the National Outcome Scale for Children and Adolescents (HoNOSCA)	2 sections, 15 scales Includes disruptive, over activity, self-injury, substance misuse, scholastic or language skills, illness or disability, hallucinations and delusions, emotional, peer relationships	Clinician report; Parent/carer report; Self rated (SR)	Clinician and parent/carer report: 3-18 years	13 items plus two further optional questions in the clinician report, 5 minutes to complete.	Clinician report: 5 point scale (“no problem” through to “severe to very severe problem”)	Free of charge for UK Services	English
			Self-report: 13-18				
					Parent/carer and self-report: 5 point scale		
					(“not at all” through to “severely”)		
8. Kidscreen	KIDSCREEN-10: uni-dimensional global HRQoL. KIDSCREEN-27 – 5 dimensions: Physical Well-Being, Psychological Well-Being, Autonomy & Parents, Peers & Social Support, School Environment. KIDSCREEN-52 – 10 dimensions: Physical Well-being, Psychological Well-being, Moods and Emotions, Self-Perception, Autonomy, Parent Relations and Home Life, Social Support and Peers, School Environment, Social Acceptance (Bullying), and Financial Resources	Measures are primarily child report with a proxy measure for parent/carers.	8-18 years	10, 27 or 52 items	5 point scale, labels vary	Use of the questionnaires is free for research purposes but the KIDSCREEN manual must be purchased	A range of versions have been translated into over 25 different languages
9. Pediatric Symptom Checklist (PSC)	The Pediatric Symptom Checklist (PSC) and the Youth Pediatric Symptom Checklist (Y-PSC) are parent/carer- and child-report questionnaires designed for screening school-age children for psychosocial problems. It assesses both emotional and behavioural problems. All items are summed to give an overall score of psychological impairment	The Pediatric Symptom Checklist (PSC) and the Youth Pediatric Symptom Checklist (Y-PSC)	PSC: 6-16 years	35 items in both versions	3 point scale (never, sometimes, often)	Free	Available in Japanese, English and Spanish
			Y-PSC: 11 years +	17 item version also available			
10. Strengths and Difficulties Questionnaire (SDQ)	25 closed-ended questions making up 5 subscales: conduct symptoms, emotional symptoms, hyperactivity, peer relationships and prosocial behaviour. It has an additional impact supplement, which assesses the extent to which problems have had an impact on aspects of the child’s life.	Parent/carer, teacher and self-report versions.	Parent/carer and teacher reports: 4-16 years	25 items (5 minutes)	0, 1, 2 (not true, somewhat true, certainly true)	Paper copies can be used for free	A range of versions have been translated into over 70 different languages
			Self-report: 11-17				
11. Youth Outcome Questionnaire (YOQ)	Covers six key areas: intrapersonal distress, somatic, interpersonal relations, critical items, social problems, behavioural dysfunction	A parent/carer report outcome and tracking measure	Parent/carer report: 4-17 years	64 items or 30 items	5 point response scale	Yes	English, Dutch, French, Korean, Spanish, and Swedish
		A youth self-report outcome and tracking measure	Self-report: 12-18 years				
		A 30-item, single-subscale, self- report or parent/carer report outcome and progress tracking measure					

**Table 3:** Psychometric properties of 11 retained measures

**Measure**		** Validity**		**Reliability**	
	**Content**	**Discriminant**	Concurrent*	**Internal consistency**	**Test-retest**
1. Achenbach System of Empirically Based Assessment (ASEBA) [4]	Procedure for selecting items included literature review, consultation with mental health professionals and special educators and pilot testing with parents/carers, teachers and youth	CBCL: Discriminates between referred and non-referred samples	CBCL: DSM IV checklist 0.49-0.87; clinical diagnoses 0.27-0.6; CPRS-R 0.71-0.8, BASC PRS 0.52-0.89; TRF: CTRS-R 0.77-0.89; BASC TRS 0.46-0.87	CBCL: 0.63-0.97	CBCL: 0.82-0.94 (8 days)
				TRF: 0.72-0.97	TRF: 0.6-0.95 (16 days)
				YSR: 0.55-0.95	YSR: 0.68-0.91 (8 days)
		TRF: Discriminates between referred and non-referred samples			
		YSR: Discriminates between referred and non-referred samples			
2. Beck Youth Inventories (BYI) [25]	Pilot studies used to select initial items based on verbal reports of children who were in therapy, distribution of responses and the ability of an item to differentiate between clinical and non-clinical sample.	Discriminates between clinical group and matched controls; children seeing SEN services and matched controls.	CDI 0.26-0.72; RCMAS scales 0.13 - 0.7; PHCSCS scales 0.06 - 0.67; CASS:S 0.27 - 0.73	0.86-0.92	0.63-0.89 (1 week median)
3. Behavior Assessment System for Children (BASC) [26-28]	Multiple sources (teachers, students, psychologists, psychiatrists) were asked to write operational definitions of the constructs. Items were written to agree with definitions.	TRS: Discriminates between different clinical profiles PRS: Discriminates between different clinical profiles SRP: Discriminates between different clinical profiles	TRS: SSRS 0.03-0.6	TRS: 0.82-0.90	TRF: 0.81-0.96 (1 month)
			PRS: CBCL 0.71-0.84, SSRS 0.02-0.62	PRS: 0.74-0.80	PRS: 0.70-0.85 (1 month)
				SRP: 0.80-0.82	SRP: 0.64-0.86 (1 month)
			SRP: MMPI (0.78-0.89)		
4. Behavioural and Emotional Rating Scale (BERS-2) [29,30]	Detailed rationale for content and format of existing subscales (derived based on consultation, item and factor analysis) and rationale for the new career strength subscale. 2. Validity of items checked with classical item analysis used to choose items. 3. Differential item functioning analysis to reinforce and show lack of bias in items.	TRS: Discriminates between normative sample and sample with emotional and behavioural problems. Scales can discriminate between students without disabilities, with learning disabilities and behavioural disorders PRS: Discriminates between normative sample and sample with emotional and behavioural problems.	TRS: WMSSCSA 0.29 - 0.85; SSBD 0.26-0.80; SAED 0.25 -0.71; SSRS 0.21 -0.73; TRF 0.27 - 0.75	TRS: 0.84 - 0.98	TRS: 0.85-0.99 (2 weeks); 0.53-0.68 (6 months)
		YRS: Discriminates between normative sample and sample with emotional and behavioural problems.	PRS: CBCL 0.09 - 0.91; SSRS 0.43 – 0.79	PRS: 0.84 - 0.97	PRS: 0.82-0.92 (2 weeks)
			YRS: YSR 0.03-0.81; SSRS 0.32-0.73	YRS: 0.79 - 0.95	YRS: 0.84-0.91 (2 week)
5. Child Health Questionnaire (CHQ) [31-33]	Items & concepts compared with other published child and adolescent health assessment measures such as CHQ, CHIP etc.	Parent 50: Discriminates between clinical and normative groups	Parent 50: HUI 0.29-0.58	Parent 50: 0.66 -0.94	Parent 28: 0.14-0.78
		Parent 28: Discriminates between clinical and normative groups	Parent 28: VAS rating of Health 0.15-0.5	Parent 28: 0.75	
				Child Form: 0.62 - 0.94	
6. Child Symptom Inventory-4 (CSI-4) [34]	Based on DSM-IV	Parent Checklist: Discriminates between normative and clinical sample	Parent Checklist: CBCL 0.01- 0.73	Parent Checklist: 0.74- 0.94	Parent Checklist: 0.46-0.87 - Symptom severity scores; 0.34-0.83- symptom count scores (Average 4.3 weeks)
			Teacher Checklist: TRF 0.08- 0.73	Teacher Checklist: 0.71 -0.96	
		Teacher Checklist: Discriminates between normative and clinical sample			
					Teacher Checklist: 0.47-0.88- Symptom severity scores; 0.54-0.84- symptom count scores (2 weeks)
7. Health of the Nation Outcomes Scales for Children and Adolescents (HoNOSCA) [35-37]	Based on HONOS(adults), consultation to adapt usage to children and adolescents	Clinician report: Discriminates between in-patients and outpatients Self-Rated: Discriminates between in-patients and outpatients	Clinician report: CGAS 0.64, SDQ (PR)0.4, PCS 0.62, Behaviour Checklist 0.44		Clinician report: r = 0.69 (6 months, for cases recognised as unchanged); SR: r = 0.81 (1 week)
			Parent/carer report: SDQ (PR) = 0.32		
			Self-report: SDQ = 0.66		
8. Kidscreen [38-41]	Kidscreen 52: Literature reviews, expert consultation (Delphi Method), children’s focus groups, card sort technique piloted with 8-18 year olds. Methods from Item response theory (IRT) and classical test theory used to reduce number of items to 52.	Kidscreen 52: Discriminates between healthy and mentally or physically ill children.	Kidscreen 52: KINDL scales 0.16-0.68; Peds QL 0.44-0.61	Kidscreen 52: 0.77-0.89	Kidscreen 52: 0.56-0.77(2 weeks)
			Kidscreen 27: Peds QL 0.16-0.54; CHIP 0.39-0.62; YQOL-S 0.37-0.63		
			Kidscreen 10: PEDSQL 0.57; CHIPS 0.63; YQOL-S 0.61		
	Kidscreen 27: Derived from Kidscreen 52 using EFA, Mokken Scale analysis, Rasch partial credit modelling, MAP analysis and CFA.				
				Kidscreen 27: 0.78-0.84	Kidscreen 27: 0.61-0.74 (2 weeks)
		Kidscreen 27: Discriminates between healthy and mentally or physically ill children.			
				Kidscreen 10: 0.82	Kidscreen 10: 0.7 (2 weeks)
	Kidscreen 10: IRT and differential item functioning techniques were used to reduce 27 items to ten items.				
		Kidscreen 10: Discriminates between healthy and mentally or physically ill children.			
9. Pediatric Symptom Checklist (PSC) [42-46]	The scale is a shortened and revised form of the Washington Symptom Checklist.	Parent/carer report: PSC:	Parent/carer report: PSC: CGAS 79-92%, к =0.82; CBCL к =0.52; DICA к = 0.74; PSC-17: CIS 0.74; CGAS 0.64; CBCL 0.60	Parent/carer report	Parent/carer report: PSC: 0.86 (1 week) Youth report: 0.45 (4 months)
		Discriminates between referred and non-referred children and children with and without problems.		PSC: 0.89	
				PSC-17: 0.79-0.89	
	PSC-17: Cross validated factor analysis on PSC.				
		PSC-17:Discriminates between children with and without diagnoses(ADHD, externalising, depression) Youth report: Discriminates between students identified as having attentional/behavioural problems and those without these problems			
			Youth report: CDI к =0.47; RCMAS к =0.42; Teacher rating of attentional and behavioural problems к =0.58		
10. Strengths and Difficulties Questionnaire (SDQ) [6,47,48]	Not reported.	Parent/carer report: Discriminates between clinical and normative populations	Parent/carer report: CBCL, 0.59-0.87	Self-report: 0.69-0.82	Self-report: 0.21-0.62 (4-6 months)
				Parent/carer report: 0.63-0.85	Parent/carer report: 0.57-0.72 (4-6 months)
				Teacher report: 0.7-0.88	Teacher report: 0.65-0.82 (4-6 months)
11. Youth Outcomes Questionnaire (YOQ) [49,50]	Aided by having adolescents define content of data, additional items from adolescent health/welfare experts and reviews of lit	Youth report: Discriminates between clinical and community samples Parent/carer report: Discriminates between clinical and community samples	Youth report: KINDL 0.73, CDI 0.58	Youth report: 0.77-0.96	Youth: 0.74-0.85 (1 week)
					Parent/carer: 0.8 (average 3 weeks)
				Parent/carer report: 0.92	

*Only magnitude, not sign, of correlation reported.

NB, no evidence was found of any measure being tested for bias or differential performance in different UK populations (e.g., ethnic, regional or SES differences or for any measure being successfully used to drive up performance within services). These categories are not included in the table to aid clarity but did form part of the initial range of considerations.

*Abbreviations*: *CASS:S* Conners-Wells Adolescent Self-Report Scale: Short form, *CDI* Children’s depression Inventory, *CGAS* Children's Global Assessment Scale, *CHIP* Child Health and Illness Profile, *CPRS-R* Revised Connors Parents Rating scale, *CTRS-R* Revised Connors Teacher Rating Scale, *HUI* Health Utilities Index, *PedsQL* Pediatric Quality of Life Inventory, *PHCSCS* Piers- Harris Children's Self-Concept scale, *PCS* Paddington Complexity Scale, *RCMAS* Revised children’s manifest Anxiety Scale, *SAED* Scale for Assessing Emotional Disturbance, *SSBD* Systematic Screening for Behaviour Disorders, *SSRS* Social Skills Rating System, *VAS* Visual Analogue Scale, *WMSSCSA* Walker-McConnell Scale of Social Competence and School-Adjustment-Adolescent Version, *YQOL-S* Youth Quality of Life surveillance version instrument.

## Discussion

This paper represents the first review that evaluates existing broadband measures of child and parent reported mental health and wellbeing outcomes in children, in terms of both psychometrics and implementation. The eleven measures identified (1. Achenbach System of Empirically Based Assessment (ASEBA), 2. Beck Youth Inventories (BYI), 3. Behavior Assessment System for Children (BASC), 4. Behavioral and Emotional Rating Scale (BERS), 5. Child Health Questionnaire (CHQ), 6. Child Symptom Inventories (CSI), 7. Health of the National Outcome Scale for Children and Adolescents (HoNOSCA), 8. Kidscreen, 9. Pediatric Symptom Checklist (PSC), 10. Strengths and Difficulties Questionnaire (SDQ), 11. Youth Outcome Questionnaire (YOQ)) all have potential for use in routine practice. Below we discuss some of the key properties, strengths and limitations of these measures and outline practice implications and suggestions for further research.

In terms of acceptability for routine use (including burden and possible potential for dissemination) three of the measures identified, though below the stipulated half hour completion time, were in excess of fifty items (ASEBA, BASC, the full BYI) which might limit their use for repeated measurement to track change over time in the way that many services are now looking to track outcomes [3]. These measures are most likely to be useful for detailed assessments and periodic reviews. In addition the majority of the measures require license fees to use, introducing a potential barrier to use in clinical services. Kidscreen, CHQ, SDQ, HoNOSCA and PSC are all free to use in non-profit organizations (though some only in paper form and some only under particular circumstances).

In terms of scale properties, all the measures identified have met key psychometric standards. Each of the final measures has been well validated in terms of classical psychometric evaluation. In addition, a range of modern psychometric and statistical modelling approaches have also been applied for some of these measures item response theory (IRT) methods, including categorical data factor analysis and differential item functioning, e.g. [51]. This is particularly true for the Kidscreen, which is less well known to mental health services than some of the other measures identified. However, analyses carried out for this measure include both Classical and IRT methods [38].

All measures were able to provide normative data and thus the potential for cut off criteria and to differentiate between clinical and non-clinical groups. However, we found no evidence of any measure being tested for bias or differential performance in different ethnic, regional or socio-economic status (SES) differences in the UK. Sensitivity to change evidence was only found for YOQ, ASEBA and SDQ, which were found to have the capacity to be used routinely to assess change over time [52]. The other measures may have such capacity but this was not identified by our searches. However, it is worth noting that many of the measures used a three-point Likert scale (e.g., PSC, SDQ). This may result in limited variability in the data derived, possibly leading to issues of insensitivity to change over time and/or floor or ceiling effects if used as a measure of change. In terms of impact of using these measures, we found no evidence that any measures had been successfully used to drive up performance within services.

In terms of implications for practice it is hoped that identifying these measures and their strengths and limitations may aid practitioners who are under increased pressure to identify and use child- and parent-report outcome measures to evaluate outcomes of treatment [12].

Some limitations should be acknowledged with respect to the current review. It is important to note that some measures were excluded from the current review purely because they did not fit our specific criteria. These measures may nevertheless be entirely appropriate for other purposes. In particular, all measures pertaining to specific psychological disorders or difficulties were excluded because the aim of the review was to identify broad measures of mental health. We recognize that many of these measures are psychometrically sound and practically useful in other settings or with specific groups. Furthermore, as recognized by Humphrey et al. [53], in their review of measures of social and emotional skills, we acknowledge that the publication bias associated with systematic reviews is relevant to the current study and may have affected the inclusion of measures at the final stage of the review. However, we maintain that this criterion is important to ensure the academic rigor of the measure validation.

In terms of future research what is required is more research into the sensitivity to change for these and related measures [54,55], their applicability to different cultures and, the impact of their use us as performance measurement tools [56]. Research is also needed on the impact of these tools on clinical practice and service improvement [57]. In particular in the light of clinician and service user anxiety about use of such tools [58-60] it would be helpful to undertake further exploration of their acceptability directly with these groups (Wolpert, Curtis-Tyler, & Edbrooke-Childs: A qualitative exploration of clinician and service user views on Patient Reported Outcome Measures in child mental health and diabetes services in the United Kingdom, submitted).

## Conclusions

Using criteria taking account of psychometric properties and practical usability, this review was able to identify 11 child self-report mental health measures with the potential to inform individual clinical practice, feed into service development, and to inform national benchmarking. The review identified some limitations in each measure in terms of either the time and cost associated with administration, or the strength of the psychometric evidence. The different strengths and weaknesses to some extent reflect the heterogeneity in purposes for which mental health measures have been developed (e.g., estimation of prevalence and progression in normative populations, assessment of intervention impact, individual assessment at treatment outset, tracking of treatment progress, and appraisal of service performance). While it is anticipated that as use of such measures diversifies the evidence base will expand, there are some gaps in current knowledge about the full range of psychometric properties of many of the shortlisted measures. However, current indications are that the 11 measures identified here provide a useful starting point for those looking to implement mental health measures in routine practice and suggest options for future research and exploration.

## Abbreviations

ASEBA: Achenbach system of empirically based assessment; ASI: Adolescent symptom inventory; BASC: Behavior assessment system for children; BERS: Behavioral and emotional rating scale; BYI: Beck youth inventories; CAMHS: Child and adolescent mental health services; CASS:S: Conners-wells adolescent self-report scale: short form; CBCL: Child behaviour check list; CDI: Children’s depression inventory; CGAS: Children's global assessment scale; CHIP: Child health and illness profile; CHQ: Child health questionnaire; CPRS-R: Revised connors parents rating scale; CSI: Child symptom inventories; CTRS-R: Revised connors teacher rating scale; ECI: Early childhood inventory; HoNOSCA: Health of the national outcome scale for children and adolescents; HUI: Health utilities index; PCS: Paddington complexity scale; PedsQL: Pediatric quality of life inventory; PHCSCS: Piers- Harris children's self-concept scale; PRS: Parent report scale; PSC: Pediatric symptom checklist; RCMAS: Revised children’s manifest anxiety scale; SAED: Scale for assessing emotional disturbance; SDQ: Strengths and Difficulties Questionnaire (SDQ); SES: Socio-economic status; SR: Self rated; SRP: Self-report of personality; SSBD: Systematic screening for behaviour disorders; SSRS: Social skills rating system; TRF: Teacher report; TRS: Teacher report scale; VAS: Visual analogue scale; WMSSCSA: Walker-McConnell scale of social competence and school-adjustment-adolescent version; YI: Youth’s inventory; YOQ: Youth outcome questionnaire; Y-PSC: Youth pediatric symptom checklist; YSR: Youth self report; YQOL-S: Youth quality of life surveillance version instrument.

## Competing interests

The initial review reported in this paper was jointly funded by the Department of Health (DH) and the Department for Children, Schools and Families (DCSF, now the Department for Education). Further work extending the review and developing the paper was funded by the DH Policy Research Programme. This is an independent report funded by DH. The views expressed are not necessarily those of the Department. Authors have no other interests (financial or otherwise) relevant to the submitted work.

## Authors’ contributions

JD, developed the review protocol, led the initial literature review and drafted the article in full. TC and JB independently provided advice on the psychometric evidence for the review process and provided independent appraised of the final 11 measures selected for detailed review. PF advised on the review process, and revised and commented on the drafting of the paper. PP carried out secondary searches for the detailed review of the final 11 measures, summarized the information derived and populated the tables relating to implementation features and psychometric properties. MW led the initial project commissioned by DH and DCSF, contributed to the drafting of the paper and provided overall sign off of the final draft. All authors read and approved the final manuscript.
